# Iatrogenic acute aortic dissection induced by off-pump coronary artery bypass grifting: A case report and review of the literature

**DOI:** 10.1097/MD.0000000000009206

**Published:** 2017-12-22

**Authors:** Jiachen Li, Xinliang Guan, Ming Gong, Xiaolong Wang, Hongjia Zhang

**Affiliations:** a Department of Cardiac Surgery, Beijing Aortic Disease Center, Beijing Anzhen Hospital, Capital Medical University; b Beijing Institute of Heart Lung and Blood Vessel Diseases; c Beijing Lab for Cardiovascular Precision Medicine; d Beijing Engineering Research Center of Vascular Prostheses, Beijing, China.

**Keywords:** iatrogenic acute aortic dissection, off-pump coronary artery bypass grifting

## Abstract

**Rationale:**

Iatrogenic acute aortic dissection (IAAD) induced by cardiac surgery is a fatal complication, with 0.04% of therapeutic procedures and worse outcomes than spontaneous aortic dissection.

**Patients concerns:**

A 64-year-old male complaining of intermittent chest tightness for 4 years received an off-pump coronary artery bypass grifting (OPCABG) and IAAD was found during surgery.

**Diagnosis:**

Unstable angina, coronary artery triple vessel lesion, IAAD.

**Interventions:**

An ascending aorta replacement surgery was implemented immediately and extracorporeal membrane oxygenation (ECMO) was applied during surgery. The patient suffered from oliguria symptoms and began to receive continuous renal replacement therapy (CRRT) after surgery. What was worse, osteofascial compartment syndrome (OCS) was also confirmed the day after surgery.

**Outcomes:**

The CRRT and ECMO were both removed and the condition of the right leg was also stable. But the patient passed away because of uncontrollable sepsis 18 days after the surgery.

**Lessons:**

OPCABG is clearly the riskiest type of surgery associated with IAADs in cardiac surgical procedures, which should be considered with great concern. Whether ECMO should be used postoperatively in IAAD patients is still a controversial subject, due to some fatal complications linked with it.

## 1 Introduction

Iatrogenic acute aortic dissection (IAAD) is a fatal complication that may occur during coronary interventions and open cardiac surgery,^[1]^ such as complex percutaneous coronary intervention,^[2]^ off-pump coronary artery bypass grafting (OPCABG),^[3]^ thoracic endovascular aortic aneurysm repair,^[4]^ and trans-aortic valve replacement.^[5]^Among these procedures, cardiac surgery-induced aortic dissection has been estimated to occur in approximately 0.04% of therapeutic procedures, with worse outcomes than spontaneous aortic dissection.^[6]^ In this report, we present an IAAD induced by OPCABG, including the surgical treatment, postoperative management, and experimental summary.

## 2 Case presentation

**Figure 1:**
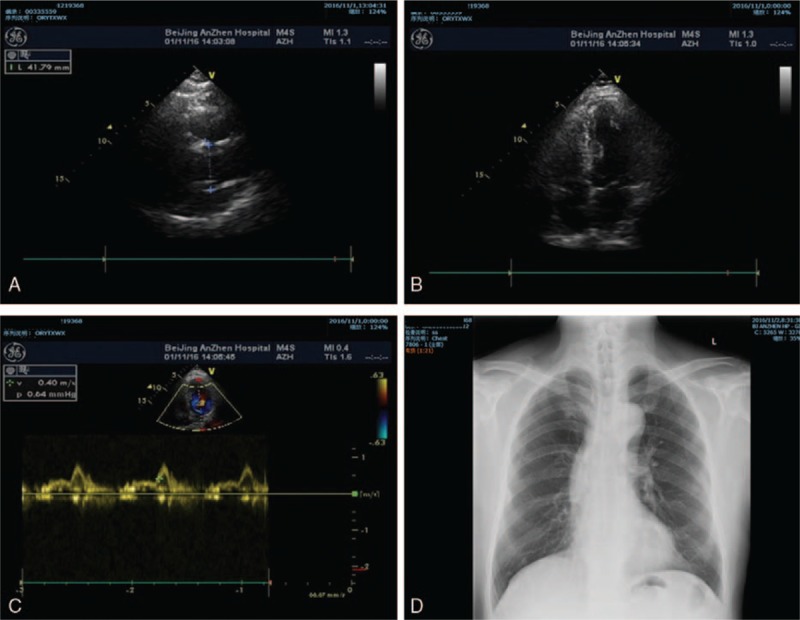
(A–C) Preoperative Doppler ultrasound heart (UCG) results, demonstrating acceptable cardiac function, with a left ventricular ejection fraction (LVEF) of 52% and no valvular regurgitation or hydropericardium. (D) Preoperative chest X-ray, no abnormality could be found in the imaging examination.

A 64-year-old male complaining of intermittent chest tightness for 4 years was admitted to the Cardiology Clinic of Beijing Anzhen Hospital, Beijing, China on November 1, 2016. He had a history of hypertension for 15 years, and his blood pressure was typically controlled at approximately 140/80 mm Hg during a resting state. He had also been diagnosed with gout in a local hospital 7 years earlier and had intermittently received medication. Otherwise, he had no medical history. After admission into the ward, the patient underwent coronary angiography (CA), which demonstrated multiple-branch coronary lesions. Following a comprehensive consultation between cardiologists and cardiac surgeons, the patient was transferred to the Department of Cardiac Surgery, Beijing Anzhen Hospital, Beijing, China on November 7, 2016. Physical examinations and routine laboratory tests indicated no abnormalities; color Doppler ultrasound heart (UCG) demonstrated acceptable cardiac function, with a left ventricular ejection fraction (LVEF) of 52% and no valvular regurgitation or hydropericardium, the chest X-ray also showed no abnormality (Fig. 1).

**Figure 2:**
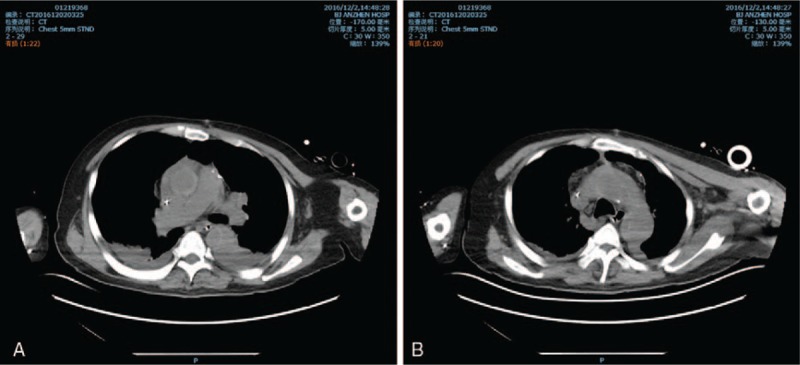
(A and B) The patient's postoperative aorta CT result, the CT aortography (CTA) was not practiced because of his renal dysfunction. Stanford A aortic dissection could be clearly confirmed by this CT scan.

An operation was performed on November 14, 2016 at Anzhen Hospital, Beijing, China; the patient underwent grafting of the left internal thoracic artery to the left anterior descending coronary artery and sequential grafting of the saphenous vein to the left circumflex branch, diagonal branch, and descending posterior branch. This procedure went smoothly during bypass grafting, and the mean flows were satisfied. However, when the proximal anastomotic bleeding was assessed, an ascending aorta hematoma was identified, and aortic dissection was rapidly diagnosed. The cardiopulmonary bypass (CPB) was then immediately established via right atrium and right femoral artery cannulation; a dissection was specifically indicated when the aorta was incised, and the innominate artery was involved by the dissection when exploring the aorta arch. Based on a comprehensive review of the patient's coronary condition and the risks associated with hypothermic circulatory arrest, ascending aorta replacement surgery was the final decision. Following the ascending aorta replacement, the reassessed mean flows of the grafts were good; however, the blood pressure consistently remained at a low level of 60 to 80/40 to 50 mm Hg, and the oxygen saturation became untenable during the removal process of the extracorporeal circulation (ECC). The process of auxiliary circulation continued for approximately 40 minutes and did not work well. Eventually, extracorporeal membrane oxygenation (ECMO) was applied with right axillary artery and right femoral vein cannulation. After the surgery, which lasted approximately 9.5 hours, the patient was returned to the intensive care unit (ICU) with the assistance of ECMO. Volume supplementary, transfusion, cardiotonics, and diuretics were pointedly applied after surgery. During the initial 12 hours after surgery, the patient underwent severe coagulopathy, and the blood pressure remained at 60/45 mm Hg with an ECMO flow of approximately 3.7 L/minute. After the patient's condition was evaluated, an exploratory thoracotomy surgery was performed the same afternoon; substantial blood clots were identified inside the pericardium during the operation. After the surgery, the blood pressure increased to 85 to 90/65 to 70 mm Hg with the same flow supported by ECMO. On the ninth day after the initial surgery, the patient demonstrated an alternative arrhythmia condition of atrial fibrillation and left bundle branch block. The cardiologist was requested for consultation and implanted a temporary pacemaker via the jugular vein; a normal sinus rhythm gradually recovered in approximately 4 hours. The cardiac function of the patient gradually improved with the assistance of ECMO, and the LVEF recovered from 20% on the first day after surgery to 45% on the 12th day after surgery. On the 14th day after surgery, ECMO was removed, and the blood pressure consistently remained at approximately 120/80 mm Hg, with a normal sinus rhythm of approximately 75 beats/minute. The patient subsequently underwent aorta CT, and Stanford A aortic dissection was definitively confirmed (Fig. 2).

During the next morning of the exploratory thoracotomy surgery, the patient suffered from oliguria symptoms and began to receive continuous renal replacement therapy (CRRT). The patient's anuria condition lasted for 12 days with anuria or oliguria symptoms, and the blood urea nitrogen (BUN) and creatinine levels decreased by degrees. On the 13th postoperative day, the patient's urine volume became acceptable, the BUN and creatinine levels returned to normal, and the CRRT was gradually removed. The muscle tone and circumference of the patient's right shank increased during the afternoon after the exploratory thoracotomy surgery, his skin temperature became wet-cold, and the pulse in the dorsal artery of the foot became weakened; the orthopedist was requested for an urgent consultation, and the diagnosis of osteofascial compartment syndrome (OCS) was confirmed. An emergent incision and decompression surgery of the osteofascial compartment was implemented at bedside. Debridement and suturing surgery was conducted 6 days later.

**Figure 3:**
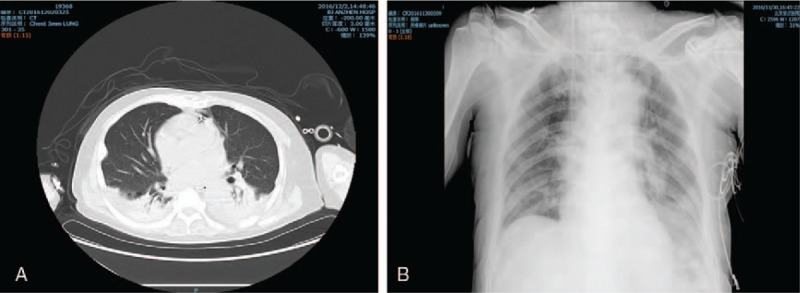
(A) The postoperative chest CT scan, and (B) the postoperative bedside chest radiograph. There was evidently exudative lesion and lung consolidation in bilateral lung tissue.

Unfortunately, when the CRRT and ECMO were both removed and the condition of the right leg was also stable, an uncontrollable infection occurred. In fact, the infection was considered to be of substantial importance from the outset. Routine laboratory tests showed that the white blood count (WBC) was acceptable before the 5th day after surgery; however, it abruptly increased from 16.91 to 28.72 G/L on the 6th day and subsequently remained at an extremely high level. The core temperature appeared normal due to concealment by CRRT and ECMO. The sputum culture on the 11th day after surgery showed a small amount of fungal growth, the catheter tip culture indicated capitate gram-positive cocci, and the blood culture was negative. The reassessed sputum culture on the 14th day after surgery showed a small amount of gram-positive cocci and gram-positive bacilli, and the blood culture was negative. At midnight on the 15th day after surgery, several hours after ECMO removal, the patient's core temperature surged from 37.0 to 39.2°C, and the fever condition subsequently continued. Different types of antibiotics were pertinently used; however, the effectiveness was poor. A daily bedside chest radiograph and chest CT scan were also obtained, and there was evidently exudative infection and consolidation in the lung (Fig. 3). Throughout the postoperative stage, the patient was in the unconsciousness state. Finally, the patient passed away because of uncontrollable sepsis 18 days after the surgery.

## 3 Discussion

### 3.1 IAADs in open heart surgery

Most IAADs induced by thoracotomy are accompanied by complicated lesions of the cardiovascular system, such as 3 or more coronary branch lesions and chronic heart failure caused by valvular heart disease.^[4]^ In a retrospective study from Switzerland several years ago,^[7]^ 10 patients (0.12%) presented with IAAD among 8624 cardiac surgical procedures under CPB, including 6 coronary artery bypass grafts (CABG), 1 aortic valve replacement (AVR), 1 AVR and CABG, 1 mitral valvuloplasty (MVP) and CABG, and 1 ascending aorta replacement surgery. Among these cases, 1 patient developed lethal tamponade as a result of aortic rupture before surgery. Postoperatively, 6 patients responded well, and 3 patients died (33%), including 2 patients with severe postanoxic encephalopathy and 1 patient with severe postoperative cardiogenic shock. In another retrospective study from Austria, 7 patients sustained an IAAD in 3000 open heart cases during a 3-year period (2002–2004), which resulted in an incidence of 0.23%, including 3 MVR, 2 CABG, 1 Bentall (ascending aortic replacement with aortic valve replacement) and 1 single lung transplantation with ECMO support.^[8]^

### 3.2 IAADs associated with OPCABG

We determined that CABG was clearly the riskiest type of surgery associated with IAADs in cardiac surgical procedures. Compared with coronary artery bypass grafting performed with ECC, the risk of aortic dissection may be increased in OPCABG; in a retrospective analysis of acute ascending aortic dissections that complicated coronary artery bypass grafting surgery in 3031 patients,^[9]^ IAAD occurred in 3 patients among 308 patients operated on without ECC (0.97%) and 1 patient among 2723 patients operated on under ECC (0.04%). This difference was statistically significant (*P* < .001). In summary, IAADs induced by OPCABG are rare but potentially fatal complications of open heart surgery, and it remains difficult to clarify the reason and standard treatment. Nevertheless, aorta calcification and atheromatous plaque formation caused by advanced age and side-clamping of the aorta during OPCABG surgery are the main risk factors. The prevention of AAD with the appropriate means requires standard practice in cardiac surgery. When AAD occurs, an immediate diagnosis and interposition graft should occur to enable a better prognosis.

### 3.3 Surgical approaches for IAADs and postoperative ECMO support

The standard surgical approach for IAAD should be ascending aorta replacement surgery, which has been widely accepted and approved by the previously described retrospective studies.^[7,8]^ ECMO has been one of the most useful life-saving therapies for patients with unstable hemodynamics despite optimal loading and a maximal dose of an inotrope medication during the previous 2 decades,^[10]^ particularly for acute refractory cardiogenic shock and impaired oxygenation. Several cases of acute aortic dissection successfully treated by postoperative ECMO have been reported,^[11,12]^ and a recent pilot study has indicated that thoracic acute aortic dissection (TAAD) patients supported by postoperative ECMO exhibit a long-term survival comparable to patients who did not receive ECMO.^[13]^ However, whether it is worth the effort to use ECMO as a rescue therapy in critically ill patients remains an argument in the field because of its numerous complications. Research has indicated that nosocomial infection is a fatal complication linked with ECMO: among 75 ECMO patients, 20 patients developed NI (infection rate 26.7%); 58 pathogens were isolated, including 43 strains of gram-negative bacteria (74.1%) and 15 strains of gram-positive bacteria (25.9%). Multidrug resistant strains were highly concentrated.^[14]^ In our case, the patient also suffered and passed away from severe sepsis, which was out of control with the use of ECMO. Moreover, OCS was a major complication associated with ECMO that has been identified in nearly 30 cases in our center after the application of ECMO. In our case, although OCS was perceived and treated in a timely manner, the myoglobin and creatinine levels substantially increased, which critically impaired the previously weakened kidney function. In our opinion, the use of ECMO remains controversial in IAAD patients; as these patients suffer from multiple preoperative cardiac high risks, the length of ECMO use is not foreseeable, which may be a deadly problem.
